# Views on sick-listing practice among Swedish General Practitioners – a phenomenographic study

**DOI:** 10.1186/1471-2296-8-44

**Published:** 2007-07-30

**Authors:** Malin Swartling, Stefan Peterson, Rolf Wahlström

**Affiliations:** 1 Department of Neurosciences, Rehabilitation Medicine, Uppsala University, Uppsala, Sweden; 2 Department of Public Health and Caring Sciences, Family Medicine and Clinical Epidemiology, Uppsala University, Uppsala, Sweden; 3 Department of Public Health Sciences, International Health (IHCAR), Karolinska Institutet, Stockholm, Sweden

## Abstract

**Background:**

The number of people on sick-leave started to increase in Sweden and several other European countries towards the end of the 20^th ^century. Physicians play an important role in the sickness insurance system by acting as gate-keepers. Our aim was to explore how General Practitioners (GPs) view their sick-listing commission and sick-listing practice.

**Methods:**

Semi-structured interviews with 19 GPs in 17 Primary Health Care settings in four mid-Sweden counties. Interview transcripts were analysed with phenomenographic approach aiming to uncover the variation in existing views regarding the respondents' sick-listing commission and practice.

**Results:**

We found large qualitative differences in the GPs' views on sick-listing. The sick-listing commission was experienced to come either from society or from patients, with no responsibility for societal interests, or as an integration of these two views. All the GPs were aware of a possible conflict between the interests of society and patients. While some expressed feelings of strong conflict, others seemed to have solved the conflict, at least partly, between these two loyalties.

Some GPs experienced carrying the full responsibility to decide whether a patient would get monetary sick-leave benefits or not and they were not comfortable with this situation. Views on the physician's and the patient's responsibility in sick-listing and rehabilitation varied from a passive to an empowering role of the physician.

GPs expressing a combination of less inclusive views of the different aspects of sick-listing experienced strong conflict and appeared to feel distressed in their sick-listing role. Some GPs described how they had changed from less to more inclusive views.

**Conclusion:**

The clearer understanding of the different views on sick-listing generated in this study can be used in educational efforts to improve physicians' sick-listing practices, benefiting GPs' work situation as well as their patients' well-being. The GP's role as a gatekeeper in the social security system needs further exploration. Our findings could be used to develop a questionnaire to measure the distribution of different views in a wider population of GPs.

## Background

Physicians' sick-listing practice is one determinant for the level of sick-listing [1]. The number of people on sick-leave benefits in Sweden more than doubled between 1997 and 2002 without evidence of a corresponding increase in morbidity [2]. Other western European countries showed a similar pattern [3]. The number of sick-days has slowly declined in Sweden, after peaking in April 2002, partly due to a simultaneous increase in disability pensions [4]. This high level of sick-leave, strains the national budget, and probably induces negative effects for the patients [5,6].

Physicians are gate-keepers in the sickness benefit system in Sweden, as well as in most other western European countries. They mediate between the patients' needs and demands and the formal rules [7]. The certifying physician has an "expert role" [1] to determine to which extent disease or injury is impairing the patients' ability to  perform their work, while the case manager at the local social insurance office formally determines whether the patient is entitled to monetary sickness benefits.

Sick-listing practices have been shown to vary between individual physicians [8,9], physicians of different specialities [9,10], and depend on the sex of the doctor [9] and patient [9,11]. The reason for this variation is not well known [1]. However, the patient's wish or demand for sick-listing has been suggested to influence the physician's decision to sick-list [11,12], even in cases where signs [12], and the physician's own judgement [11] speak against sick-listing. Physicians also experience difficulties in relation to sick-listing decisions [1,13-15] Reports have indicated that sick-listing has even been perceived as a work environmental problem [16].

It is difficult to estimate how frequent the sick-listing task is among General Practitioners (GPs) in Sweden and other European countries, since different measures have been used [16]. However, about 40% of sick-listing certificates in Sweden have been shown to be issued by GPs [10] and about 60% of the GPs' report consultations including sickness certification six or more times per week [17]. Not much is known on how GPs perceive their sick-listing commission and good sick-listing practice. Increased understanding of variations of views on sick-listing among GPs' may have implications for continuing medical education and information programmes [18,19] targeted to improve sick-listing practices.

Our aim was to identify and describe the different existing views among Swedish GPs on sick-listing practice and on the sick-listing commission.

## Methods

### Data collection

A strategic sample of 19 GPs was recruited from 17 Primary Health Care Centres (PHCC) in four mid-Swedish counties via post and/or e-mail after identification through the telephone directory, and checking of age in the national list of licensed physicians. Selection criteria were sex, age and location of the GPs' place of work. Five participants were below 45 years (3 men), eight were 45–54 years (4 men), and six were older than 54 years (3 men). Eight of the PHCCs were situated in towns of more than 85,000 inhabitants, three in smaller towns of 14–27,000 inhabitants and eight in municipalities of less than 9,000 inhabitants. In total 29 GPs were asked to participate. Three men and five women declined participation or could not be reached, and two women could not find time within the data collection period.

Interviews were performed in November 2003 – July 2004 by the first author (MS) only, to avoid reliability problems related to using different interviewers [20]. The interviews took place at the informant's practice (12 GPs), in his or her home (3) or at the university department (3), depending upon each GP's preference. One interview was carried out over the phone due to illness. A semi-structured interview guide with open questions was used (Table 1 and Additional File 1), designed to focus on the GP's own experience of sick-listing and to give descriptions of concrete situations. Probing questions were used if needed to help the GP elaborate and reflect.

**Table 1:** Interview Guide

**Opening wordings for exploration of views:**
GENERAL
What comes to your mind when you hear the word "sick-listing"?

PRACTICE
Please tell me about a recent patient where sick-listing was considered
- a case where you feel comfortable about the way you handled it?
- a case where you feel less comfortable about the way you handled it?

THE COMMISSION
How do you look upon your sick-listing commission?

THE IDEAL
When sick-listing, do you ever feel you would like to act in a different way than you do, and in that case why and how?

OBSTACLES
Is there anything making it more difficult for you when trying to sick-list the way you would prefer, and in that case, what?

CONCLUDING GENERAL
Is there anything else you would like to add about sick-listing or any aspect of sick-listing?

For a complete interview guide with probing questions [see additional file 1]

The interviews took 21 to 60 minutes (median 43), were audio tape recorded after verbal consent, and transcribed verbatim. Transcripts were corroborated against the tapes. NVivo qualitative software 2.0 was used for data handling. Only the interviewer knew the participating GPs by name. They were coded throughout the analysis.

### Analysis

A phenomenographic analysis [21] was performed for three domains. Two of them (views on the sick-listing commission and on sick-listing practice) were already in focus during the interviews, while the third (views on the responsibility for sick-listing and rehabilitation) emerged during the analysis. In addition, the level of experience of conflict between the patients' and society's interest as expressed by each GP was assessed, as well as the GPs' expressions of distress or wellbeing in the sick-listing situation.

The analysis was carried out by a medical doctor (MS), and a GP and health system researcher with previous experience of phenomenographic analysis (RW). All transcripts were carefully read by these two researchers and the most significant statements in each transcript regarding a domain (area of discourse on a certain topic or phenomenon) were selected by one of them to give a short but representative version of the entire dialogue on that domain.

These short versions were then independently compared, by these two authors, to find similarities and differences that could justify grouping into different 'categories of description' or views, which were then compared [21]. For all domains the initial agreement was high (for 15–18 of the 19 GPs). Most often the disagreements were slight and could be resolved through further clarification of each analyst's understanding of the statements. If uncertainty still existed regarding where to place a statement, it was discussed with a public health physician (SP). SP also categorised a sample of other selected statements as a means to check the accuracy of our categorisation [20]. Disagreements were resolved through discussions, with reference to the total material in the transcript [22]. Revisions were often necessary to reach consensus. Working in this manner towards informed consensus is suggested as a means of assuring accuracy of analysis [22]. This procedure was repeated for each of the domains of interest. For each domain an individual GP was only attributed to one category of description or view. Such views often form a hierarchy where some views are composed of fewer aspects, compared with views higher in the hierarchy where several aspects are included [23]. We have used the term 'inclusive views' of these more complex categories of description and have presented our results in order of such inclusiveness [24]. Additionally we have also analysed how views within different domains were combined in individual GPs.

In the results section a few quotes from relevant parts of the interviews are presented to illustrate different categories. It should be noted that such excerpts usually cannot include all the aspects of a category description.

Uppsala University Ethics Committee approved the study (Dnr 03-507).

## Results

### I) Variations of views within three domains of sick-listing

#### 1. The sick-listing commission

'Commission' is here used in the meaning of a task *entrusted *to someone. Four different views on the sick-listing commission were identified (1a-1d), as were different levels of perceived conflict between the patient's interests and those of society's. Examples of individual GP's transition from one view of the commission to another and from conflict to less or no conflict are also presented here, as are reflections on the physician's role as a medical expert.

##### 1 a) Patient's Commission – certification

The sick-listing commission is limited to issuing a sickness certificate. The sick-listing commission comes from the patient. Societal interests are not the sick-listing physician's concern, nor is the responsibility for rehabilitating patients back to work. Society is understood to be opposed to the patient and physician.

##### 1 b) Patient's Commission – rehabilitation

The sick-listing commission comes from the patient. Rehabilitating the patient back to work is perceived as part of the commission. This view is thus more inclusive than the view above. However, it should be noted that, as in 1 a), this view does not include responsibility for societal interests.

##### 1 c) Society's Commission

The sick-listing commission comes from society. Following the rules, guarding or distributing society's resources in a fair way, or fulfilling the intentions of the policy-makers constitutes the commission. Rehabilitation of the patient is perceived as an integrated part of the sick-listing commission.

"I have society's commission of course. It is a politically decided benefit we have, to sick-list under certain circumstances which are decided by the authorities. We have to follow the rules given by authorities." (Dr D)

##### 1 d) Integrated Commission

The sick-listing commission is understood to come both from the patient and from society. Rehabilitating the patient back to work is understood to be in the patient's, as well as in society's interest and thus part of the sick-listing commission. This commission includes the views presented in both category 1 b and 1 c.

"If you are allowed to work with it [sick-listing] the way I consider medically correct, I don't quite see the conflict between these two commissions. Because it's somehow like using drugs; if you prescribe penicillin for tonsillitis you are doing the right thing, but if you prescribe penicillin for a cold, you're not." (Dr A)

### Conflicting commissions, the medical expert and transitions

All the GPs, irrespective of view on the sick-listing commission, were aware of a possible conflict between society's interests and those of the patients. Some expressed experiencing strong conflict, especially those with commission 1a), while others had partially solved the conflict, and some GPs, all female, seemed to have fully solved the conflict, either by taking the patients' side and not caring about societal interests (1b) or by sticking closely to society's rules (1c) or by integrating the interests of the patient and of society (1d).

GPs, who reflected on their role as a medical expert in relation to authorities, shared experiences of carrying the full responsibility to decide whether a patient would get monetary sick leave benefits and were unhappy about feeling left by social insurance officers with this task. One GP described trying to act only as an expert leaving the decision on benefits to be taken by social security authorities.

Some GPs described how they over time had changed their way of working with sick-listing, from the Patient's-, via Society's-, to the Integrated Commission, and from experiencing conflict to experiencing less or no conflict. No GP described changes in the opposite direction.

"Well when I started working here I kind of saw myself as a patient promoter in a way. [...] Like an advocate. [...] Now I am more and more beginning to see myself as an official civil servant who has to distribute tax money in a fair way. [...] It is a continuous conflict, but I see myself more as a representative of the authorities than I did before."(Dr F)

"I have changed my way of work very much [...]. Society wants me not to sick-list at all and the patient wants me, at least from the start, to sick-list as long as possible. But I think I have managed to make a fairly good synthesis out of this." (Dr E)

#### 2. The sick-listing practice

Four different ways of understanding sick-listing were found. More complicated cases that included also psychosocial aspects of sickness were mostly the focus in the GPs' reflections, while obvious biomedical cases with a clear reason for work incapability were mentioned to a much lesser extent.

##### 2 a) Sick-listing as issuing a certificate

Sick-listing is understood to be the issuing of a certificate. Sick-listing is commented on without reference to good or bad practice, and without reflection of one's own actions. Alternative ways of sick-listing are not recognised. Sick-listing is used while waiting for somatic investigations, for patients to heal by themselves, or when patients say they cannot work.

"Yeah, I haven't thought much of any other ways of sick-listing than the way I do it now. I don't know how I would perform it differently." (Dr N)

##### 2 b) Changing work situation

Beyond issuing a certificate, good sick-listing is understood as helping the patients to change their work situation. Changing the private life situation is not considered in the scope of possible objectives.

"It must be possible to adapt jobs to the individual, rather than the opposite." (Dr B)

##### 2 c) Changing lifestyle

Beyond issuing a certificate, good sick-listing is understood as helping the patients to change their lifestyles to better cope with the demands of work. Changing the work-situation is not considered.

*"She is recommended physiotherapy regularly, and exercise at the physiotherapists [...]. Try to eat better, live better, simply to have the [physical] qualifications to do this job." *(Dr M)

##### 2 d) Holistic view

Good sick-listing is to recognise the illness or injury hindering the patient from work, in a perspective of both the working and the private life situation. Good sick-listing practice includes addressing all identified problems, in order to rehabilitate the patient's working capacity. This inclusive view embraces the two views above (2b, 2c).

"But the right thing is that you get support and help to change yourself so that you can handle your own work situation and your family situation." (Dr E)

#### 3. Responsibility for sick-listing and rehabilitation

Five different views were found regarding *who *is carrying *which *responsibility in relation to sick-listing and rehabilitation back to work. *How *the physicians handle their perceived responsibility is also included in the descriptions below.

##### 3 a) The Passive

Physicians have no responsibility for the patients' rehabilitation back to work, nor do they have any responsibility to make sure the patients themselves take on such responsibility. The physician allows the patients' ideas of what is possible in *their *case to determine the sick-listing, seemingly disregarding their own judgement of suitable measures. GPs holding this view experience strong conflict and express feelings of distress. They seem to have very few tools to handle the sick-listing situation.

"I always ask if they could consider working at all. I always assume they can, but it's not often they are of the opinion they can do that. One should perhaps get in contact with employers more, because many patients say 'It is not possible, because the employer won't accept it.' And that I don't know. I don't call the employer to check." (Dr C)

##### 3 b) The Protecting

It is the physician's responsibility to solve the patients' problems, including getting patients out of unpleasant or financially difficult situations. The patients' own responsibility is not apparent. In case the physician and patient have different views on the need of sickness certification the physician lets the patient decide or tries to compromise.

"...included in my assignment is to do what I think is good for my patient. When somebody comes as my patient I want to take care of his or her problems in the best possible way." (Dr Q)

##### 3 c) The Authoritative

The physician's responsibility is to lead and outline a strategy: pushing the patient towards rehabilitation measures, or trying to withhold sick-listing when that is considered better. The patients' responsibility is understood to follow the GP's "prescription". When the physician and the patient make different judgements on the need for sickness certification the physician compromises.

*"...before you sick-list you somehow explain why you sick-list and for how long, and if it is part-time or full time. [...] At times you should perhaps avoid dithering too much, but be quite firm." *(Dr M)

##### 3 d) The Supporting

The responsibility for the sick-listing and rehabilitation is shared by the physician and the patient. The GPs should interact actively and supportively with the patient during rehabilitation efforts. When the physician and patient have different opinions on the need for sickness certification, the physician compromises or follows her own judgement making her own decision.

*"I even think I was the one bringing sick-listing up; 'Could this be a way... that you can get out of this during a limited time?' [...] at the first encounter I bring this up that this is something I see as something very temporary." *(Dr O)

##### 3 e) The Empowering

Physicians' responsibility is helping the patients to shoulder *their *responsibility for their own sick-listing, rehabilitation and whole life-situation. If disagreement between the physician and the patient occurs on the need for sickness certification the physicians compromise or follow their own judgement making their own decision.

"I write it down, because they don't remember [...] otherwise: Find out the name and telephone number of – one: your case manager at the social security office, two: your immediate boss and, three: the occupational health service. [...] And simply by doing something, a process starts in people. Nearly all of them actually do it." (Dr K)

"Because most people say immediately something like: 'No, that's not possible at my work-place.' and 'It will never work.' [...] I say: 'That's what all employers say, but it's not like that and I'm not allowed to take that into consideration."' (Dr A)

### II) Combinations of views

In the last stage we analysed how the presented views within different domains were combined for individual GPs (Table 2). Moving from the least inclusive 'Passive' view (3a) to the most inclusive 'Empowering' view (3d) on the responsibility for sick-listing and rehabilitation, the GPs successively hold more and more inclusive views also of the *other *aspects of sick-listing.

**Table 2:** Relations between GPs' views on different aspects of sick-listing

**Responsibility for sick-listing and rehabilitation**	**Sick-listing commission**	**Sick-listing practice**	**ID**
(3a) Passive	(1a) Patient- certification	(2a) Issuing a certificate	C
	Patient- certification	Issuing a certificate	J
	Patient- certification	Issuing a certificate	N
(3b) Protecting	Patient- certification	Issuing a certificate	P
	(1b) Patient- rehabilitation	(2b) Changing work situation	B
	Patient- rehabilitation	Changing work situation	G
	Patient- rehabilitation	(2d) Holistic view **	Q
(3c) Authoritative	(1c) Society	(2c) Changing life-style	M
	Society	(2b) Changing work situation	L
(3d) Supporting	(1b) Patient- rehabilitation	(2d) Holistic view **	T
	(1c) Society	(2b) Changing work situation	F
	Society	(2d) Holistic view **	O
	(1d) Integrated *	Holistic view	H
	Integrated	Holistic view	R
	Integrated	Holistic view	S
(3e) Empowering	(1b) Patient- rehabilitation	Holistic view	K
	(1c) Society	Holistic view	D
	(1d) Integrated *	Holistic view	E
	Integrated	Holistic view	A

(1a) etc. refers to view 1a etc. as numbered in the text.

ID = physician identification

* Integrating society's as well as the patients' interests into one sick-listing commission.

** Sick-listing as exploring and taking as many aspects as possible into account, i.e., work-related factors as well as life-style.

GPs with the 'Passive' view on responsibility have the same combination of the least inclusive views on *all *domains: they view the sick-listing commission to be a 'Patient's commission' (1a) and understand sick-listing as limited to issuing a sickness certificate. They see no alternative ways of sick-listing (2a) and experience strong conflict between the patient's and society's interests and feel distressed in the sick-listing situation (3a). At the other extreme we find the GPs with an 'Empowering' view of the responsibility on sick-listing and rehabilitation, where two of the GPs hold the most inclusive view of *all *aspects of sick-listing (1d, 2d, 3e). Between these extremes we found a gradient towards more and more inclusive views.

## Discussion

We have described qualitatively different views on sick-listing among these Swedish GPs. GPs with a combination of the least inclusive views experienced strong conflict and seemed more distressed in the sick-listing situation than others. The sick-listing commission was seen to come either from the patient or from society or from both.

All interviewed GPs were aware of a possible conflict between society's and patients' interests. Some GPs in our study, all women, seemed to have resolved the conflict of this double role, although by different means. GPs in Scotland also described a conflict between their "advocate role and their role as a judge" [25]. Among Norwegian physicians 55% reported that they deliberately had written favourable disability pension certificates as seen from the patient's perspective at least once per year and 11% reported that they did so monthly or more often regarding sickness certification [26].

GPs' viewing themselves as the de facto decision-maker on eligibility for sick-leave benefits, were uncomfortable with this situation. It has been shown in a Swedish study that social insurance officers, instead of using their own judgment, accept the recommendation of the physicians as final [27], which could be part of the experiential background related to this view. We question how GPs can be accepted to function as gate-keepers in the social security system if they see their sick-listing commission to come from the patient only. The social security officers may need strengthening as case managers for the "expert role" to function as intended.

Five different views, from 'Passive' to 'Empowering', were found regarding responsibility of sick-listing and rehabilitation. Similar differences in views on how to relate to patients with asthma have been shown [28]. It was also shown in a British research report that differences in GPs' perspective on their role in sickness absence management affect their involvement in their patients' rehabilitation back to work. The 'non interventionist' GP characterised in that report resembles our 'passive' view of the responsibility of sick-listing and rehabilitation, and in several respects their 'firm negotiator' resembles the 'empowering' view in our study [29]. Our finding that the 'empowering' GPs view their role to aid the patients to shoulder *their *responsibility implies that patients have a responsibility, whether they take it on or not. This is in line with previous findings [28], but contrasts with some literature on patient centeredness where it is argued that the patients should be *invited *by the physician to take as much responsibility as they want [30,31].

The role of leadership, management and guidelines was conspicuously absent from the aspects the GPs expressed as important for good sick-listing. However, lack of leadership on all levels in the health care system has been pointed out as one problem for optimal sick-listing [16].

The GPs views were less or more inclusive with respect to the comprehensiveness of the aspects of sick-listing they took into account. GPs with more inclusive views would have more options [24] to handle sick-listing situations, which may be beneficial for them and their patients, similar to what has been shown in another professional context, where engineers with a broader understanding of their work were judged by their colleagues to be more competent in performing their task [23]. GPs with the least inclusive views, the 'Passive', seemed to feel out of control, experienced strong conflict and expressed distress in the sick-listing situation. They probably need support to adopt other, more inclusive views to improve their sick-listing practice, not only for their patients' sake, but also for their own wellbeing, in line with the demand-control theory [32]. We suggest that such support could take the form of exposing the doctor to other views in a learning situation arranged for reflective interaction.

Some GPs described how they had changed from less inclusive to more inclusive views. Similarly, it has been described that a widespread opinion among British GPs was that it is easy to move up and down a continuum of 'approaches' to sickness certification (strictly medical reasons in one end and multiple factors, including non-medical reasons in the other, and a third approach in between) and how some GPs expressed that they have moved positions over the course of their career, while others felt that they vary approaches depending on the situation of individual patients [29]. This may seem to be in conflict with our findings, but having a more inclusive view does not implicate that all sick-listing cases are approached taking all aspects into consideration at each occasion. Therefore, our findings do not exclude the described 'movements' depending on the individual patient.

It has been shown that individual GPs can change views after an educational intervention [19]. For such a change to occur, the learner's existing conceptions must be challenged [21], calling for interactive educational strategies [33,34]. Understanding GPs' views of sick-listing could be helpful in developing interventions aiming at improving their sick-listing practices. In our view, good sick-listing practices are characterised by reaching the optimal balance between high medical benefits for the individual, and minimised negative effects both for the individual, and for the society.

### Methodological considerations

This study is based on interviews with 19 GPs. Several studies have shown that "each phenomenon, concept or principle can be understood in a limited number of qualitatively different ways" [35], and that 15–20 informants normally capture the existing variation in views and experiences in a homogenous group of people [22,23]. In our analysis we found that all but one of the views were shared with at least one other GP, which gives support to the assumption that we have reached an acceptable level of saturation regarding existing views.

By using the phenomenographic approach we aimed at getting a deeper illumination of the GPs' understanding of phenomena related to sick-listing, beyond stated attitudes, which have since long been known to show ambiguous relations to behaviour [36]. Consequently, in a Norwegian study, no relation could be found between GPs' sick-listing behaviour and their attitudes [37]. In a more recent Norwegian survey, GPs' relationship to sick-listing was assessed [38]. Three groups, distinctly different from the majority of the GPs, were distinguished. Their group B, one out of two groups with a positive attitude to sick-listing for psychosocial reasons, felt burdened and permitted patients to decide to a large extent, and resembles the GPs holding the "passive" view of sickness certification in our study. GPs with this passive view described sick-listing for psychosocial reasons, but we do not know their attitude to doing so if it would be measured by the same question as in the Norwegian study. Our assumption is that the description of categories or views as in our study may have a closer relationship to practice behaviour [24,39] than attitudes, although we cannot present any proof of such a relationship in this study. This limitation is shared with other qualitative methods.

How different views combine in individual informants has not commonly been presented or discussed in phenomenographic articles. We could see a pattern in the way the views on the different domains were distributed within individual GPs, which could indicate how more inclusive views might develop step by step. To become more conclusive, these aspects need more exploration as our focus was not on how views had developed over time and, therefore, other interpretations are possible.

The trustworthiness of findings should be illuminated in qualitative studies [40]. By asking the GPs to present their own descriptions of managing authentic sick-listing cases, we received material that was close to their actual practice and where their way of presentation to a great extent reflected their views. Through analysing this material in a structured way, we believe that a reasonable degree of credibility has been reached. In particular, the dual categorisation by two researchers and the negotiated consensus lend support to this assumption [41]. The transferability of our findings to other contexts in Sweden could be assumed to be reasonably high as the situation for sick-listing GPs is similar all over the country and as we interviewed doctors in different types of environment. However, transfer of our findings to other countries, should be done with greater caution.

The consistency of our findings can be questioned as changes in the social environment and the regulations regarding sick-listing may influence practice. However, in our view, the categories of description are at a level of interpretation that is not directly influenced by such external changes. Quantification of different views among GPs and the extent to which differences in views are reflected in outcomes of patient care would be useful.

## Conclusion

The clearer understanding of the different views on sick-listing generated in this study can be used in educational efforts to improve physicians' sick-listing practices, thereby benefiting GPs' work situation as well as their patients' well-being. The indicative sex differences in handling the conflict between patients' and society's interests could warrant further exploration. So does the GP's role as a gatekeeper in the social security system. Our findings could also be used to develop a questionnaire to measure the distribution of different views in a wider population of GPs, which would make it possible to find associations between views and GP characteristics.

## Competing interests

The author(s) declare that they have no competing interests.

## Authors' contributions

MS coordinated the study, did all the interviews, corroborated the transcripts against the tapes and drafted the manuscript. MS and RW read all the transcripts and did the analysis. SP read a sample of the interviews, and helped in the analysis. RW and SP commented on the manuscript. All authors conceived of the study, participated in its design, read and approved the final manuscript.

## Pre-publication history

The pre-publication history for this paper can be accessed here:



## Supplementary Material

Additional file 1 Complete interview guide. The complete set of questions and probing questions used to guide the interviewer during the interviews. Click here for file
