# Supplementary material for: Views on sick-listing practice among Swedish General Practitioners – a phenomenographic study
Source: BMC Fam Pract. 2007 Jul 30;8:44. doi: 10.1186/1471-2296-8-44 (PMC1988796; doi:10.1186/1471-2296-8-44)
Supplement: Additional file 1 — Complete interview guide. The complete set of questions and probing questions used to guide the interviewer during the interviews. [file 1471-2296-8-44-S1.doc]

**INTERVIEW GUIDE**

# GENERAL

1a) What comes to your mind when you hear the word ‘sick-listing’?

PRACTISE – HAPPY

2a) Please tell me about a resent patient where sick-listing was considered – a case where you feel happy about the way you handled it?

(Do you have any other example where longer time sick-listing was at risk?)

2b) What was your **goal** with this sick-listing?

- In general what is your goal when sick-listing?

2c) Which **alternatives** did you consider when deciding whether sick-listing this patient or not?

- In general how do you look upon different alternatives when sick-listing?

2d) What did you tell the patient about the actual sick-listing, in this case?

- What do you normally say about the sick-listing, when prescribed?

2e) What is your view of the role of the patient in this case of sick-listing?

PRACTICE - LESS HAPPY

3a) Please tell me about a recent patient where sick-listing was considered – a case where you feel less happy about the way you handled it?

(Do you have any other example where longer time sick-listing was at risk?)

3b) What was your **goal** with this sick-listing?

- In general what is your goal when sick-listing?

3c) Which **alternatives** did you consider when deciding whether to sick-listing this patient or not?

- In general how do you look upon different alternatives when sick-listing?

3d) What did you tell the patient about the actual sick-listing, in this case?

- What do you normally say about the sick-listing, when prescribed?

3e) What is your view of the role of the patient in this case of sick-listing?

4a) The two examples you have given, are they representative of your day to day experience, when it comes to sick-listing?

THE COMMISSION

5a) How do you look upon your commission to sick-list?

5b) On whose commission do you feel you are acting?

5c) Who gave you this commission?

How do you weigh the health benefits of sick-listing against the risk of unwanted side effects?

THE IDEAL

6a) When sick-listing, do you ever feel you would like to act in a different way than you do, and in that case why?

6b) In what way would you like to act differently?

OBSTACLES

7a) Is there anything making things more difficult for you when trying to sick-list the way you would prefer? If yes, which?

CONCLUDING GENERAL QUESTION

8a) Is there anything else you would like to add about sick-listing or any aspect of sick-listing?

*Questions a) posed to all interviewed GPs.*

*Probing questions b)-e) asked when more information was wanted.*
